# Polymorphisms of the *OXTR* gene explain why sales professionals love to help customers

**DOI:** 10.3389/fnbeh.2013.00171

**Published:** 2013-11-27

**Authors:** Willem Verbeke, Richard P. Bagozzi, Wouter E. van den Berg, Aurelie Lemmens

**Affiliations:** ^1^Department of Business Economics, Erasmus School of Economics, Erasmus University RotterdamRotterdam, Netherlands; ^2^Department of Marketing, Ross School of Business, University of MichiganAnn Arbor, MI, USA; ^3^Department of Marketing, University of TilburgTilburg, Netherlands

**Keywords:** oxytocin, *OXTR*, sales professionals, genomic imaging, social salience, connectivity, translational implications, Granger causality

## Abstract

Polymorphisms of the *OXTR* gene affect people's social interaction styles in various social encounters: carriers of the *OXTR* GG, compared to the *OXTR* AA/AG in general, are more motivated to interact socially and detect social salience. We focus on sales professionals operating in knowledge intensive organizations. Study 1, with a sample of 141 sales people, shows that carriers of the *OXTR* GG allele, compared to the *OXTR* AA/AG allele, are more motivated to help customers than to manipulatively impose goods/services on them. Study 2, using genomic functional magnetic resonance imaging (fMRI) on a sample of 21 sales professionals processing facial pictures with different emotional valences, investigates key nuclei of social brain regions (SBRs). Compared to *OXTR* AA/AG carriers, *OXTR* GG carriers experience greater effective connectivity between SBRs of interest measured by Granger causality tests using univariate Haugh tests. In addition, the multivariate El-Himdi and Roy tests demonstrate that the amygdala, prefrontal cortex, and pars opercularis (inferior frontal gyrus) play key roles when processing emotional expressions. The bilateral amygdala and medial prefrontal cortex (mPFC) show significantly greater *clout*—influence on other brain regions—for GG allele carriers than non-carriers; likewise, the bilateral pars opercularis, left amygdala, and left mPFC are more receptive to activity in other brain regions among GG allele carriers than AG/AA allele carriers are. Thus, carriers of the *OXTR* GG allele are more sensitive to changes in emotional cues, enhancing social salience. To our knowledge, this is the first study on how insights from imaging genetics help understanding of the social motivation of people operating in a professional setting.

“We're not in the coffee business serving people,” Howard Behar, the former president of Starbucks, told me. “We're in the people business serving coffee. Our entire business model is based on fantastic customer service. Without that, we're toast.”

(Duhigg, 2012, p. 141).

## Introduction

What actually motivates employees in firms like Starbucks, Walt Disney, or IBM to interact prosocially and pleasantly with customers and crave to develop positive relationships with them? For instance, “What Walt (Disney) really wanted were employees with ready smiles and a knack for dealing pleasantly with larger numbers of people” (Bright, 1987, p. 111). This capacity to be friendly and deal pleasantly with customers is known as emotional labor (Ashforth and Humphrey, 1993). Here, we conceive emotional labor not as an amodal cognitive decision (“I decided to be friendly with my customer”), but as changes in the neuroendocrine functioning of oxytocin (OT), a process which occurs largely unconsciously and affects people's social interactions such as inferring emotions of others and working to promote mutuality (Bethlehem et al., 2013). For example, administering OT helps people to behave cooperatively and generously and has thus been called the “moral molecule” (Zak, 2009). In what follows, we explore the role of OT levels in employees interacting in business contests. Specifically, we examine whether sales professionals who are carriers of polymorphisms of the *OXTR* gene like helping customers, and whether sales professionals who are carriers of the GG *OXTR* allele, as opposed to the GA/AA *OXTR* allele, show different brain connectivity when they process human faces with different emotional valences (e.g., Bethlehem et al., 2013).

OT is synthesized in the paraventricular nuclei (PVN) and supraoptic nuclei (SON) of the hypothalamus (e.g., Bos et al., 2012). Parvocellular neuropeptide containing neurons in the PVN and SON synthesize and project to different nuclei in the central nervous system (CNS), especially the amygdala (the central amygdala in particular), nucleus accumbens (Nac), orbital frontal cortex (OFC), medial prefrontal cortex (mPFC), and brainstem regions (Landgraf and Neumann, 2004; Ross and Young, 2009; Bos et al., 2012; Veening and Olivier, 2013). There are a vast number of mechanisms of action by which OT reaches and affects these different brain regions (see Bethlehem et al., 2013 for a good overview). As research using different technologies continues to unfold [e.g., pharmacological interventions/optogenetic activation/functional magnetic resonance imaging (fMRI)] a huge variety of OT mechanisms affecting CNS functioning will and have been observed. For instance, Knobloch et al. (2012) show that PVN-central amygdala connections are stronger than SON-central amygdala connections, but also that accessory magnocellular hypothalamic nuclei affect the central amygdala, all influencing activity of inhibitory amygdala cells, thus reducing expression of fear (see also Ross and Young, 2009). OT release is also found in plasma, extracellular and cerebrospinal fluid (Born et al., 2002; Landgraf and Neumann, 2004; Veening et al., 2010; Veening and Olivier, 2013). OT has a longer half-life than traditional neurotransmitters, which allows it to travel over longer distances to different brain regions (Landgraf and Neumann, 2004; Veening and Olivier, 2013). In addition, depending on the brain areas involved, different cascading effects of neuro peptide release could be found (Landgraf and Neumann, 2004; Bethlehem et al., 2013).

In this paper we build (and focus) on earlier observations that higher OT levels change the connectivity between different brain regions and affect social motivation and the detection of social salience (Skuse and Gallagher, 2009; Meyer-Lindenberg et al., 2011; Bethlehem et al., 2013). Studies using OT administration show different changes in connectivity, especially between the amygdala and such brain nuclei such as the OFC, anterior cingulated cortex (ACC), hippocampus, and precuneus, where they become more connected when participants are confronted with social-emotional laden stimuli (e.g., Riem et al., 2011; Feldman, 2012; Bethlehem et al., 2013).

Social motivation entails a desire (wanting) and enjoyment (liking) to feel included in a social relationship or group, and social salience involves a proclivity to attend to facial expressions that are relevant to feeling accepted by others (Chevallier et al., 2012). Two factors affect how OT influences social motivation and social cognition: contextual effects and individual differences (Bartz et al., 2011).

First, in cooperative environments, but not in contrary (opposing) environments, OT affects the amygdala—a key hub in neural networks involved in social cognition (Adolphs, 2010)—and guides attention through its connectivity to the fusiform gyrus and several regions in the prefrontal cortex to focus scrutiny on biologically relevant stimuli from other people, such as eye movements and other facial cues. This facilitates social salience [“Are others (still) favorable to me?”] and selective attention to changing valences (positive, neutral, negative) of facial expressions that are relevant to social relationships (Adolphs, 2001; Skuse and Gallagher, 2009). OT also affects the NAc (which has dense dopamine and OT receptors) that through its connectivity with the mPFC is involved in decision utility estimation, allowing computation of salience value (“Is this social cue rewarding?”) (Adolphs, 2008; Aragona and Wang, 2009; Meyer-Lindenberg et al., 2011; Chevallier et al., 2012). Finally, OT affects the mPFC, and through the mPFC activation, people consciously experience a companion as “enjoyable,” (subjective hedonic value) which then affects social memory for that specific person (Depue and Morrone-Strupinsky, 2005, p. 340). These coupled neural processes explain “wanting” to be included in a group or to create social relationships and thus spontaneous initiation of actions toward others (e.g., approaching or helping others) (Aragona and Wang, 2009; Kohls et al., 2013). During social interaction, people shape their social skills and empathic abilities; for instance, this is when the mirror neuron system undergoes plasticity to form new connections between neurons, which in turn facilitate actions that lead to social inclusion (Iacoboni, 2009).

Besides the neuropeptide OT, endogenous opioids (a peptide) also affect the processes related to social motivation and social cognition (Chevallier et al., 2012, p. 233). Facilitatory effects of the opioids are thought to be exerted from the hypothalamic arcuate nucleus which has fibers that reach brain regions like the mPFC, amygdala, and Nac, all known to be densely populated with opioid receptors (Mansour et al., 1988). Opioids are produced when individuals engage in social companionship, making it enjoyable (Depue and Morrone-Strupinsky, 2005, p. 340; Nelson and Panksepp, 1998; Berridge et al., 2009; Trezza et al., 2010). Given that opioids affect enjoyment (and memory of companionship and subsequent wanting processes) there is some conjecture that OT and opioid “neurochemical projections work together as part of a unitary brain process or affiliative circuit which regulates mammalian affiliative behavior” (Nelson and Panksepp, 1998, p. 466). However, the way in which OT and opioids (might) interact is still a matter of debate and beyond the scope of this paper (see Depue and Morrone-Strupinsky, 2005, p. 340).

Second, individual differences affect how OT influences the connectivity of different regions such as the Nac, amygdala, fusiform gyrus, and OFC (Tost et al., 2012). Genetic differences in the OT receptor gene *(OXTR)* have been targeted (e.g., Meyer-Lindenberg et al., 2011), as carriers of the *OXTR* GG allele show higher desire (wanting) to engage socially with others and are better in detecting social salience than carriers of the AA/AG alleles (Tost et al., 2010). Developmentally, carriers of the *OXTR* AG/AA, as opposed to the GG alleles, tend to engage in different developmental trajectories that affect their brain networks. Those less attuned to detect social salience might become less socially motivated over time (Chevallier et al., 2012).

Seeking translational implications of research on OT (meaning that OT has specific effects in definitive environmental contexts; Bartz et al., 2011), we focus on a specific context where employees (sales professionals) interact with customers. Two alternative interaction styles are commonly distinguished: customer orientation (CO) vs. selling orientation (SO). CO is a self-initiated inclination to help customers satisfy their needs, often with the hope of building long-term relationships that will benefit both seller and buyer (Zablah et al., 2012, p. 22). In contrast, SO is largely a one-sided preference to meet one's own needs primarily, often manipulatively, at the expense of the customer and with a short-run time horizon (Saxe and Weitz, 1982). We explore two questions in two consecutive studies: (1) Are sales professionals high in CO, as opposed to SO, more likely to be carriers of the OT receptor (*OXTR*) polymorphism GG as opposed to the AG/AA alleles? (2) When confronted with faces showing different emotional valences, do sales professionals who carry the *OXTR* GG, vs. AG/AA alleles, show stronger network connectivity between the social brain regions (SBRs)? The SBRs are known to be associated with social salience, and the focus is on the central role the amygdala plays in the connectivity of different nuclei within SBRs when people are confronted with faces of different emotional value or salience (Skuse and Gallagher, 2009; Bethlehem et al., 2013).

Different polymorphisms in the *OXTR* gene are located on chromosome 3p25-3p26.2, specifically the rs 53576, rs 2254298, rs 1042778, rs237887, and rs2228485, which are known to affect social functioning (Inoue et al., 2010; Meyer-Lindenberg et al., 2011). Here we focus on the single nucleotide polymorphism rs53576 on the third intron of the *OXTR* gene because recent research suggests its centrality as a modulator in social motivation and social salience (e.g., Rodrigues et al., 2009; Kumsta and Heinrichs, 2012). Phenotypically, carriers of the *OXTR* GG allele, vs. AG/AA alleles, display more prosocial behavior and empathy (see Kumsta and Heinrichs, 2012, for an overview). Carriers of the *OXTR* GG, vs. AA allele, showed higher positive affect (Lucht et al., 2009), were more dependent on reward (reliant on social approval) (Tost et al., 2010) and possessed more personal psychological resources such as self-esteem and mastery (Saphire-Bernstein et al., 2011). Parents who were carriers of the *OXTR* AA showed lower levels of sensitive responses to their toddlers (Bakermans-Kranenburg et al., 2008). Carriers of the *OXTR* AA/AG allele, as opposed to the GG, also scored lower on empathy (“reading the mind in the eyes” test) (Rodrigues et al., 2009). More important for our study, as customers ultimately are the ones who evaluate sales professionals, Kogan et al. (2011) showed that carriers of the *OXTR* GG allele, as opposed to carriers of the *OXTR* AA/AG, display more prosociality in nonverbal displays when judged by outsiders. Likewise, Tost et al. (2010) found that the former have a higher degree of reward dependency (interest in social approval). Studying carriers of the *OXTR* GG allele, vs. AG/AA, during a two-person investment game, Krueger et al. (2012) showed that the former had higher trust in the other person. In addition, American (but not Korean) carriers of the *OXTR* GG allele were more likely to seek social support when feeling distressed (Kim et al., 2010). Finally, carriers of the *OXTR* AG/AA alleles (rs53576) reveal lower empathy and lower motivation to build social bonds (e.g., Rodrigues et al., 2009; Skuse and Gallagher, 2011; Kumsta and Heinrichs, 2012), but not all studies replicated these findings (e.g., Apicella et al., 2010). We hypothesize that sales professionals operating in actual professional environments and who are carriers of the *OXTR* GG (vs. *OXTR* AG/AA) allele will more likely engage in CO than SO.

An association between polymorphisms of a candidate gene and a phenotype brings up the possibility of uncovering implicit neural mechanisms that might explain this epidemiological finding. For example, Domes et al. (2007) show that the trust effect of mutuality can be explained by the fact that OT improved people's ability to accurately infer emotions from others [see also Bartz et al. (2010) for the effect of oxytocin on empathy].

Endophenotype research using fMRI on the differences between carriers of the *OXTR* GG, vs. AG/AA, may indicate that both carriers have developmental trajectories affecting the structure and the connectivity between brain nuclei of networks involved in social salience (e.g., Meyer-Lindenberg et al., 2011). For instance, carriers of the *OXTR* AA/AG, compared to GG carriers, show morphometric differences of the hypothalamus and amygdala (decreased volume in hypothalamus using voxel based morphology for the AA/AG carriers), and increased structural connectivity between the hypothalamus and both the dorsal anterior cingulate gyrus and the amygdala for the GG carriers (Tost et al., 2010). Tost et al. (2010) also found that carriers of the *OXTR* GG, as opposed to the *OXTR* AA/GA, have an increased connectivity between the hypothalamus and the amygdala during processing of emotionally salient social stimuli. Given that carriers of the *OXTR* GG are more prone to seeking social interaction, they should train their empathic and social understanding abilities accordingly (Kohls et al., 2012). A type of training and learning of the SBR is believed to occur in a Hebbian-like learning, as neurons that connect the different nuclei in the SBR show more connectivity when confronted with emotional stimuli (Iacoboni, 2009).

We conceive the SBR as including the amygdala, insula, pars opercularis, premotor cortex, and mPFC. The amygdala is included because of its key role in detecting social salience (Adolphs, 2008), and the insula is included because it is known to be involved in perception of emotional cues with different valences, as well as processing conflicting valences (happy, disgusted, etc.) (Singer et al., 2008; Bastiaansen et al., 2011; Riem et al., 2011). The mPFC and the pars opercularis are included because they are densely connected with the amygdala, and both play an important role in detecting social salience (Ghashghaei et al., 2007; Adolphs, 2008; Skuse and Gallagher, 2009). Viewing emotional facial expressions comes with associated facial mimicry and triggers an increase of activity in the precentral motor face area of the observer (Van der Gaag et al., 2007; Bastiaansen et al., 2011). Hence, the precentral motor face area is included in the SBR we investigated.

Adolphs (2008) and Skuse and Gallagher (2009) suggested that the amygdala plays an important role in social cognition (functioning as a hub), as it is responsible for processing salient stimuli, such as occurs when positive- and negative-valenced stimuli are processed (Adolphs, 2010). This central role of the amygdala occurs because it is densely connected with different nuclei such as the inferior occipital gyrus (part of the pars opercularis) and the mPFC. Therefore, in our study we particularly explored the connectivity of the amygdala with other SBRs while people processed pictures of faces with different emotional valence. To capture SBR activation, we explored effective connectivity between brain nuclei in brain networks (defined as the causal influence that one brain region exerts on another), as well as the causality of activation patterns during specific tasks (e.g., Bethlehem et al., 2013). We examined the effective connectivity of nuclei present in the SBR of carriers of the GG *OXTR* allele vs. the AA and AG alleles using univariate Granger causality testing in our experimental design (Haugh, 1976; Deshpande et al., 2008; Jiao et al., 2011).

Most studies have examined the existence and strength of directional relations between brain regions using univariate tests, and effective connectivity between two specific brain regions is tested one pair at a time (Goebel et al., 2003; Roebroeck et al., 2005; Bressler et al., 2008). However, given the substantial number of pair-wise directional relationships that may exist between all brain regions (e.g., for 10 brain regions, 90 possible pair-wise directional relationships have to be tested), such univariate testing approaches should not be employed alone. That would lead to inference problems (see Bauer et al., 1988). El-Himdi and Roy (1997) recommended performing multivariate Granger causality tests to investigate the existence of significant directional relationships between multiple pairs of brain regions simultaneously. Hence, we used the El-Himdi and Roy (1997) test to estimate Granger causality.

## Study 1. *OXTR* polymorphisms and association with helping customers

### Materials and methods

#### Subjects

Dutch sales professionals (*n* = 141) volunteered for a study of the role of biomarkers in professional relationships. All subjects were Caucasian, 13% women, 87% men. Of the participants, 49% had a university degree, while others were graduates of professional education programs, with, on average, 6.8 years of professional experience. All worked in knowledge intensive firms and sold engineering solutions, financial services or IT solutions.

Respondents were divided into two groups on the basis of their *OXTR* (rs53576) genotype. In the first group were individuals with two copies of the G allele (G homozygotes; *n* = 71; 50.4%). In the second group were individuals with both one copy of the A allele and G allele [A heterozygotes (A/G); *n* = 53; 37.6%] and two copies of the A allele [A homozygotes (A/A); *n* = 17; 12.1%]. We made this division because the A allele is the dominant allele for this specific genotype, while the G allele is the recessive allele. The genotypes were in agreement with the Hardy-Weinberg equilibrium—HWE: χ^2^_(1)_ = 1.997, *p* = 0.16.

All genotyping was performed blind to demographic and clinical data. Buccal swabs were obtained from each subject. Genomic DNA was isolated from the samples using the Chemagic buccal swab kit on a Chemagen Module 1 workstation (Chemagen Biopolymer-Technologie AG, Baesweiler, Germany). DNA concentrations were measured using the Quant-iT DNA Assay kit (Invitrogen, Breda, Netherlands). The average yield was 4 μg of genomic DNA per buccal swab sample.

#### Genotyping

The region of interest from the *OXTR* gene was the single nucleotide polymorphism rs 53576. DNA was amplified by PCR using a forward primer (5′-GCCCACCATGCTCTCCACATC-3′) and a reverse primer (5′-GCTGGACTCAGAGGAATAGGGAC-3′). Typical PCR reactions contained between 10 and 100 ng genomic DNA template, 10 pmol of forward and reverse primers, and PCR was carried out in the presence of 5% DMSO with 0.3 U of BioThermAB polymerase (GeneCraft, Munster, Germany) in a total volume of 30 μg l using the following cycling conditions: initial denaturation step of 3 min at 95 μg C, followed by 40 cycles of 30s at 95 μg C, 30s at 60 μg C, 1 min at 72 μg C and a final extensions step of 3 min at 72 μg C. To determine the A/G polymorphism, PCR fragments were sequenced using the forward primer and dye terminator chemistry (BigDye v3.1, Applied Biosystems, Foster City, CA).

#### Measures

Measures of social mutuality in commercial relationships were obtained with a CO scale containing five 7-point disagree-agree Likert items. One item correlated relatively low with another item on the scale and was deleted. Confirmatory factor analysis showed that the four positively correlated items formed a single factor: χ^2^_(2)_ = 2.71, *p* = 0.26, *RMSEA* = 0.07, *NNFI* = 0.98, *CFI* = 0.99, and *SRMR* = 0.03 (Hu and Bentler, 1999). Cronbach's alpha reliability of the items was 0.77. Examples of items include, “I try to find out what kind of product would be most helpful to a customer” and “I try to give customers an accurate expectation of what the product will do for them.” Similarly, the SO scale contained five 7-point disagree-agree Likert items. Confirmatory factor analysis showed that a single factor resulted: χ^2^_(5)_ = 4.80, *p* = 0.44, *RMSEA* = 0.00, *NNFI* = 1.00, *CFI* = 1.00, and *SRMR* = 0.02 (Hu and Bentler, 1999). Cronbach's alpha reliability of the items was 0.92. Examples include, “It is necessary to stretch the truth in describing a product to a customer,” and “I paint too rosy a picture of my products, to make them sound as good as possible.” For all items of SO and CO, see Appendix.

#### Results

The mean for CO carriers of *OXTR* AA/AG was 5.95 (*SD* = 0.84) and for the GG carriers it was 6.23 (*SD* = 0.56). A *t*-test shows that the means differ significantly: *t* = 2.33, *p* < 0.05. The mean for SO and *OXTR* AA/AG carriers was 4.01 (*SD* = 0.185) and for GG carriers, 4.09 (*SD* = 1.78). A *t*-test shows that the means do not differ: *t* = 0.26, ns. In sum, CO relates to *OXTR*, as expected. SO is unrelated to *OXTR*, as forecast.

## Study 2. uncovering activations of the social brain region using genomic imaging

### Materials and methods

#### Subjects

Healthy participants were asked to participate in a study on personality and neurological processes. Twenty-one participants [13 men, 8 women; average age 34 years (*SD* = 6.13), ranging from 21 to 46 years old] and working in industries like IT, banking and industrial firms, volunteered to take part in an emotional-valenced stimuli task. The experiment had participants view video clips of human faces with different emotional valences (positive, negative, and neutral), and DNA samples were taken with the buccal swab. In the analysis to follow, we make a distinction between two groups: carriers of AA/AG alleles (8) were one group and carriers of GG allele (13) the second group. The *OXTR* polymorphism group is in HWE (χ^2^ = 3.5, *p-value* = 0.17). All subjects were right-handed, and in accordance with the guidelines specified by the local institutional review board, all signed written informed consents. An important point to note is that fMRI data analysis and time series extraction were done independently of the genotyping. This procedure was followed to ensure minimization of (misclassification) biases.

#### Stimulus and procedures

The emotional-valenced stimuli task consists of experimental stimuli of full-face, full-color video clips of five men and five women displaying various emotional states of anger, disgust, happiness, and surprise (Bastiaansen et al., 2011). In addition, we used two types of control stimuli: video clips of the same actors with neutral faces, and video clips displaying moving geometric shapes. Thus, the four experimental conditions were (1) positive emotional expressions: joy and surprise, (2) negative emotional expressions: anger and disgust, (3) neutral expressions, and (4) moving geometric shapes. Each clip was played for 3 s in 12-second blocks. Each block presented three separate clips with 1-s inter-stimulus pauses between clips. Each block was presented to a participant 12 times in pseudo-randomized order. Each block displayed only positive, negative, or neutral emotional states, or the moving geometric shapes. Conditions were counterbalanced. This setup is similar to one frequently used in experiments assessing emotional processing regions (e.g., Van der Gaag et al., 2007; Bastiaansen et al., 2011). Since there is convincing evidence that the mirror neuron system is involved in imitation as an immediate replica of the observed motor act, we also performed a control experiment, the imitation task (IT). Based on the results of contrast analysis, Imitation vs. Observation, we concluded that every subject had normally functioning emotional processing regions, as would be expected with healthy subjects (findings available on request).

#### Data acquisition

All imaging was performed on a 3T MRI scanner (GE Healthcare, Milwaukee, USA) using a dedicated 8-channel head coil for signal reception. For anatomical reference, a 3D high-resolution inversion recovery fast spoiled gradient recalled echo T1 weighted image was acquired [echo time (TE)/repetition time (TR)/inversion time = 2.1/10.4/300 ms, flip angle = 18°, matrix = 416 × 256, slice thickness 1.6 mm with 50% overlap].

For functional imaging, we used a single-shot gradient-echo echo-planar imaging sequence in transverse orientation that is sensitive to Blood Oxygenation Level Dependent (BOLD) contrast (TR/TE 3000/30ms. 64 × 96 matrix, 2.5 mm slice thickness, 39 contiguous slices), which covered the entire brain of any given experiment participant. Acquisition time was 9 min and 36 s with a time series of 192 imaging volumes per participant (including 15 s of dummy scans that were eventually discarded).

Experiments were performed in near-total darkness with all lights turned off except for the video projection. The visual stimuli were projected from the rear of a translucent screen in front of the scanner. Subjects viewed the video projection screen via a mirror system placed on top of the head coil. Stimuli were presented to subjects by the PC-based stimulation software package *Presentation Software* (Neurobehavioral Systems, Albany, California, USA) and were triggered by the scanner to ensure precise synchronization between data acquisition and presentation of the stimuli.

#### Functional imaging data analysis

The functional imaging data were analyzed using statistical parametric mapping software (SPM8, distributed by the Wellcome Trust Centre for Neuroimaging, University College London, UK) implemented in MATLAB (Version R2010a, Mathworks, Sherborn, MA, USA). Motion correction, slice-time correction, and co-registration were done according to the methodology provided by SPM8. Brain volumes were normalized to the standard space defined by the Montreal Neurological Institute (MNI) template. The normalized data had a resolution of 2 × 2 × 2 mm^3^ and were spatially smoothed with a three-dimensional isotropic Gaussian kernel, with a full width half maximum of 8 × 8 × 8 mm^3^.

**Figure 1:**
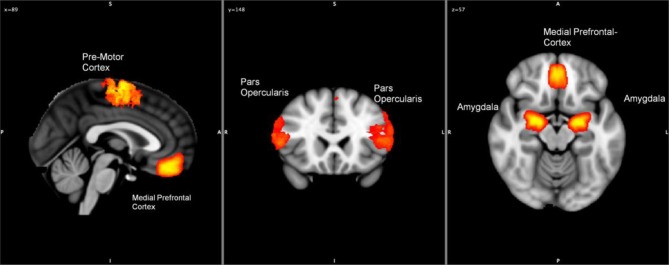
**Hypothetical locations of the regions active during the processing of emotional-valenced stimuli (amygdala, pars opercularis, motor cortex, and medial prefrontal cortex)**.

Anatomical regions of interest (ROIs) were selected based on activation in the contrast-based analysis (Emotional Faces > Neutral Faces). Using SPM8 and MarsBar (Brett et al., 2002; Martin et al., 2005; Jiao et al., 2011), the underlying time series of signal intensity were extracted from these ROI to our study (similar procedures as described in Jiao et al. (2011) and Mies et al. (2011). Granger causality analyses were performed using the ROIs time series to explore the effective connectivity of these regions active during the emotional processing task by taking into account a subject's variations of *OXTR* gene. To control for autocorrelation, a univariate or multivariate filter is applied on all the time series, for the Haugh test and El-Himdi and Roy test, respectively. The amygdala, insula, pars opercularis, mPFC and premotor cortex were selected as ROIs for the SBR (see Skuse and Gallagher, 2009). Figure 1 presents a graphical overview of the location of regions activated in the contrast analysis.

#### Genotyping

All genotyping was performed blind to clinical and demographic data and family relationships. The SNP marker rs53576 [Celera ID: C 3290335 10] is genotyped using TaqMan® SNP Genotyping Assays (Applied Biosystems, Foster City, CA, http://www.appliedbiosystems.com). TaqMan® PCR reactions were done with Universal Master Mix Amperase® UNG, 0.25 L TaqMan probe mix and 2.25 L of water for a 5 L total volume. The PCR conditions for the TaqMan® SNP Genotype Assays were: one AmpErase® step at 50.0°C for 2 min, one enzyme activation step at 95.0°C for 10 min, and 40 alternating cycles of denaturation at 92.0°C for 15 s and reannealing and extension at 58.0°C for 1 min. All PCR reactions were performed on a Perkin Elmer 9700 Thermocycler (Applied Biosystems, Foster City, CA). The fluorescence intensity of the final PCR product was measured using an LjL Analyst AD fluorescence microplate reader (LjL Biosystems, Sunnyvale, CA, http://www.moleculardevices.com) using LjL Criterion-Host Software.

#### Granger causality

To analyze the effective connectivity between the various regions of the subjects' brains, we performed univariate and multivariate Granger causality analyses where the time series under study represent the time-varying activity in the various regions of the brain for each subject. The concept of Granger causality refers to the *predictive content* conveyed by a given time series, *X*_*t*_, toward another time series, *Y*_*t*_. More precisely, *X*_*t*_ is said to Granger-cause *Y*_*t*_ if, and only if, the variance of the error in forecasting future values of *Y*_*t*_, using an optimal forecast based on the observed values of both *X*_*t*_ and *Y*_*t*_, is strictly smaller than the variance of the prediction error using an optimal forecast *only* based on the observed values of *Y*_*t*_. Therefore, the presence of a Granger causal relationship between two brain regions measures the effective connectivity between both brain regions.

The univariate analysis is based on Haugh (1976), extended to a panel data context (accounting for multiple subjects simultaneously) and, for each pair of regions, tests whether the activity in one of the regions Granger-causes activity in the other region across subjects. It therefore allows us to assess which pairs of regions are best connected to each other. In addition to the univariate tests, we performed multivariate analysis based on El-Himdi and Roy (1997) and extended to a panel data context (multiple subjects). This multivariate test considers all regions jointly and has been recently adapted by Lemmens et al. (2007) to assess whether one time series has predictive power toward *all* other time series simultaneously. Therefore, we can assess which regions are the most *influential*, that is, have the most *clout* in terms of leading to more activity in the other regions of the brain. Likewise, we can assess whether one region of the brain is more susceptible, i.e., *receptive*, to activity in other brain regions. A joint look at the clout and receptivity of the brain regions will give us a complete picture as to the degree of connectivity of each brain region, as both *clout* and *receptivity*.

#### Results

The results of the univariate Granger causality analysis using the Haugh test are shown in Table 1 for the AA and AG groups and in Table 2 for the GG group. Significant *p*-values at the 1% significance level are depicted in bold. We expected to find more significant Granger causal relationships between brain regions in the SBR network when subjects are carriers of the GG allele (Table 2), compared to the carriers of the AA and AG alleles (Table 1). Our expectations were confirmed, as Tables 1, 2 show. Participants with a GG allele of the *OXTR* gene show more univariate Granger causalities (48 significant relations) compared to the carriers of the AA and AG alleles (37 significant relations).

**Table 1:** **A/A and A/G group: Bivariate cross-ROI analysis: *p*-values for the Haugh test for testing whether ROI *i* (*i*th row) Granger-causes activity in ROI *j* (*j*th column)**.

		**ROI's**									
		**Amygdala (left)**	**Amygdala (right)**	**Insula (left)**	**Insula (right)**	**Pars. Oper. (left)**	**Pars. Oper. (right)**	**Medical prefrontal cortex (left)**	**Medical prefrontal cortex (right)**	**Premotor cortex (left)**	**Premotor Cortex (right)**
ROI's	Amygdala (left)	**−**	**−**	**0.000**	**0.000**	**0.002**	0.115	**0.000**	**0.007**	**0.000**	**0.000**
	Amygdala (right)	**−**	**−**	**0.000**	**0.000**	**0.000**	0.026	**0.000**	**0.001**	**0.000**	**0.000**
	Insula (left)	0.248	0.067	−	−	0.271	0.149	0.765	0.014	**0.003**	**0.005**
	Insula (right)	**0.007**	0.022	−	−	0.023	**0.003**	**0.006**	**0.001**	**0.002**	**0.000**
	Pars. Oper. (left)	0.262	0.011	0.265	0.703	−	**−**	0.327	0.657	0.151	0.023
	Pars. Oper. (right)	0.058	0.015	0.014	0.047	**−**	**−**	0.095	0.018	0.019	0.030
	M prefrontal cortex (l)	**0.003**	0.060	**0.001**	**0.004**	**0.000**	**0.005**	**−**	**−**	**0.000**	**0.000**
	M prefrontal cortex (r)	**0.002**	**0.000**	**0.000**	0.146	0.036	0.440	−	**−**	**0.000**	0.118
	Premotor cortex (left)	0.013	**0.005**	**0.000**	0.119	0.342	0.690	**0.010**	0.026	−	**−**
	Premotor cortex (right)	0.159	0.183	**0.004**	0.477	0.092	0.150	0.307	0.264	−	**−**

Figures in bold indicate the 1% probability level.

**Table 2:** **G/G group: Bivariate cross−ROI analysis: *p*−values for the Haugh test for testing whether ROI *i* (*i*th row) Granger−causes activity in ROI *j* (*j*th column)**.

		**ROI's**									
		**Amygdala (left)**	**Amygdala (right)**	**Insula (left)**	**Insula (right)**	**Pars. Oper. (left)**	**Pars. Oper. (right)**	**Medical prefrontal cortex (left)**	**Medical prefrontal cortex (right)**	**Premotor cortex (left)**	**Premotor cortex (right)**
ROI's	Amygdala (left)	**−**	**−**	**0.000**	**0.001**	**0.007**	**0.009**	**0.002**	**0.003**	**0.010**	**0.000**
	Amygdala (right)	**−**	**−**	**0.000**	**0.003**	**0.001**	0.060	**0.000**	**0.001**	**0.005**	0.103
	Insula (left)	**0.000**	**0.000**	**−**	**−**	0.039	0.173	**0.006**	0.142	0.031	0.137
	Insula (right)	**0.000**	**0.001**	**−**	**−**	0.042	0.045	**0.000**	**0.003**	0.031	**0.000**
	Pars. Oper. (left)	**0.001**	0.083	**0.000**	0.148	**−**	−	**0.005**	0.124	0.121	0.049
	Pars. Oper. (right)	0.020	**0.000**	**0.000**	**0.007**	−	−	0.052	**0.000**	**0.001**	**0.001**
	M prefrontal cortex (l)	**0.001**	**0.002**	**0.000**	**0.001**	**0.003**	0.207	**−**	−	0.030	0.085
	M prefrontal cortex (r)	0.014	**0.008**	**0.000**	**0.000**	**0.001**	0.294	**−**	**−**	**0.000**	0.029
	Premotor cortex (left)	0.016	**0.010**	**0.000**	**0.001**	0.012	0.012	**0.010**	0.011	**−**	**−**
	Premotor cortex (right)	0.034	**0.003**	0.011	**0.003**	0.102	0.066	0.162	0.088	−	**−**

Figures in Bold indicate the 1% probability level.

Particularly relevant are the directional relationships from the amygdala to other regions from the SBR and from the other regions to the amygdala; carriers of the AA/AG allele show less significant Granger causality than the carriers of the GG alleles. For the AA/AG allele carriers, 19 out of 32 Granger causalities are significant (Table 1), whereas for the GG allele carriers, 25 out of 32 are significant (Table 2).

**Figure 2:**
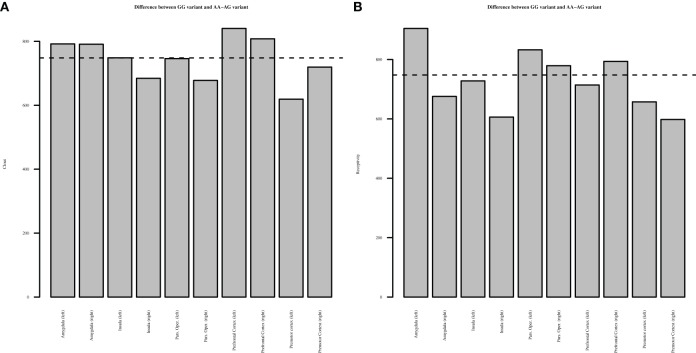
**El-Himdi Roy Test—Differences between *OXTR* GG and AA-AG allele groups.** This figure reports the test statistic that assesses whether the clout (panel **A**) or receptivity (panel **B**) of a focal brain region in the GG allele group minus the clout or receptivity of a focal brain region in the AG and AA allele groups is equal to zero. Bars exceeding the dotted line show significantly higher *clout* **(A)** or *receptivity* **(B)** for the GG allele group than for the AG and AA allele groups at a 1% probability level.

However, as mentioned earlier, the univariate Granger causality tests should be interpreted with caution as they may suffer from multiple testing biases. To test whether these results truly indicate significant differences in effective connectivity between the polymorphism groups, we employed the multivariate El-Himdi and Roy test. Figure 2 reports the results of the El-Himdi Roy tests investigating the respective clout and receptivity of each brain region toward all others. Figure 2A presents findings for *clout*, which is the ability of a brain region to Granger-cause the future activation level of another brain region. In turn, the *receptivity* of a brain region (Figure 2B) characterizes the degree to which the activity pattern in this region is predicted by prior activity in all other brain regions. In Figure 2A, we show the differences in clout between the GG and AA/AG carriers' brain regions, and Figure 2B also exhibits the differences in receptivity between the GG and AA/AG carriers' brain regions. The significant level is set to 0.01 (El-Himdi and Roy, 1997; Lemmens et al., 2007). Looking at *clout* in Figure 2A, it can be observed that only the bilateral amygdala and the mPFC clearly discriminate the carriers of the GG allele vs. the carriers of the AA/AG alleles. Likewise, *receptivity* (Figure 2B) indicates that the left amygdala, the bilateral pars opercularis and the right mPFC are discriminating regions when contrasted with the carriers of the GG vs. the AA/AG alleles.

## Discussion

Insights gained in neuroscience should have translational implications. In our research, the participants were employees in the business world selling industrial and financial solutions to professional buyers. Our findings have significant implications for practitioners, allowing us a better understanding of why some professionals demonstrate unique, functional orientations with customers, while others do not. We applied insights from neuroscience and genetics and revealed some noteworthy findings. Carriers of the *OXTR* GG, as opposed to the AA/AG, are known from basic research to have different social motivations and exhibit differences in social salience (they exhibit more social orientation, higher enjoyment in social interactions, and desire to maintain long-term relationships) (Chevallier et al., 2012). Apparently, these social motivations also spill over into a business context, where we found that GG *OXTR* carriers, compared to the AG/AA carriers, engaged in more CO. In other words, the former were more willing to help and create mutuality in sales interactions with customers. Engaging in CO is regarded as a self-initiated inclination to help customers satisfy their needs and, given that the salesperson and customer meet frequently, includes the hope to build long-term relationships. The *OXTR* GG carriers' desire to help can also be conceived as an intrinsic motivation, meaning that helping customers gives salespeople pleasure, which involves the production of OT affecting the amygdala (reducing fear) and the Nac (affecting rewards). In addition, in seeking to help and build long-term relationships with customers, salespeople create (or shape) an attractive social context that is mutually pleasant or trustworthy for both parties, thus affecting the release of OT (context).

Next, in response to emerging research suggesting that genetic variation of the *OXTR* (GG allele) gene can explain differences in the connectivity between nuclei in the brain that are relevant for social salience and related social motivation (Tost et al., 2012), we tested this relationship using both univariate (Haugh test) and multivariate (El-Himdi and Roy test) causal analyses. The El-Himdi and Roy test allows us to investigate the respective clout and receptivity of each brain area. Clout of a brain area was defined as the strength of the Granger causal relationship between one brain area and all others brain areas. In other words, the clout represents the extent to which activity in a focal brain area consistently leads to, or precedes, activity in all other brain regions. In turn, the receptivity of a brain area was defined as the strength of the Granger causal relation between all other brain regions and the focal area. Thus, receptivity represents the degree of susceptibility of a given brain area's activity to other areas' activities. Exploring the clout and receptivity of each brain region is particularly important to uncover the temporal dynamics of the SBR. It allows identification of regions that play a key role in processing emotionally valenced stimuli, and allows us to contrast these findings between subjects with different polymorphisms of the *OXTR* gene. The present study provides evidence for the identification of the regions (hubs) that discriminate between the carriers of the GG allele vs. AA or AG alleles. This test to detect connectivity in networks differs from the hypothesis-driven methods in connectivity that, for example, use the amygdala as the seed value to uncover its connectivity with other brain regions (Rilling et al., 2012).

Specifically, we focused on the way in which SBRs are effectively connected to each other when carriers of the *OXTR* polymorphisms engage in watching visual stimuli with changes in emotional valence. In both analyses, we found more effective connectivity in carriers of the GG allele, vs. AA and AG alleles. In other words, our findings suggest that there is an association between variation in the *OXTR* gene and the way in which brain regions of the SBR cohere (are effectively connected) when people process different emotional facial expressions. In particular, we found that the amygdala is important for processing emotional-valenced facial stimuli, and the amygdala responds differentially between carriers of the two GG vs. AA/AG allele groups. The higher connectivity in the SBRs of the GG carriers predisposes them to be alert and respond to social salience cues (Bethlehem et al., 2013, p. 970). First, the amygdala, compared with other regions, had more connections with other regions in the SBRs system (Haugh test), and there were more connections in the GG carriers as opposed to the AG/AA carriers. Second, using the El-Himdi and Roy test for the GG as opposed to the AA/AG carriers, we confirmed that both left and right amygdala had more clout. In other words, it influenced other nuclei of the SBR, where, for the GG as opposed to the AA/AG carriers, the left amygdala had more receptivity, meaning that more SBRs stimulated them. As Adolphs (2010, p. 48) suggested, the amygdala plays a role “in vigilance, ambiguity resolution: it modulates other brain structures to enhance the processing of stimuli about which more information needs to be acquired.”

It is also important to mention that, for the GG carriers as opposed to the AG/AA carriers, the bilateral mPFC also had more clout and the right mPFC had more receptivity. The mPFC is involved in evaluating social stimuli and also regulates the amygdala functioning, and this, in turn, leads to connectivity with other brain regions (Bachevalier and Loveland, 2006; Riem et al., 2011). Perhaps more interesting, the effects of OT on the connectivity within the SBR occur through a higher connectivity between the mPFC with other regions, especially the amygdala (Bos et al., 2012; Bethlehem et al., 2013; Sripada et al., 2013). Apparently, carriers of the AA/AG allele do not experience regulation of emotion or detect social salience as well as GG allele carriers do. Finally, the pars opercularis also showed higher receptivity for GG allele carriers, which means that they are better in detecting social salience (Skuse and Gallagher, 2009). Given the role of the pars opercularis in the MNS, and its relation to empathy, mimicry, and inferring intentions, future research should investigate more extensively how the MNS relates to the SBR and the *OXTR* in human interactions. Although this is a first step in uncovering the relationship between the *OXTR* gene and connectivity between brain nuclei (temporal dynamics) during processing of human faces with different emotional expressions, more research into the temporal dynamics of the emotional processing regions should be done to confirm our results and discover boundary conditions. Specifically, more and different ROIs could be included to uncover connectivity. Bos et al. (2012), for instance, suggested that OT might have an effect on the connectivity between OFC, anterior cingulate cortex, STS and thalamus. This is because OT is stored in large dense-core vesicles and degrades relatively slowly (only after 20 min), which boosts the potential to have effects over longer distances and affecting different regions (Bethlehem et al., 2013).

For research to have translational implications, especially to real world social contexts, it helps to better understand what it means to engage in CO as opposed to SO by sales professionals. One suggestion is that people with certain polymorphisms or dysfunctions of the OT receptor gene may feel less enjoyment in interacting with others, because OT affects both the reward system, as well as the amygdala, and amplifies opioids when a social interaction is rewarding. In the business world, as in everyday life, wanting to interact and enjoying interactions with customers might be driving forces (intrinsic motivations) to seek out and resolve interactions with customers. We all know that in business contexts, informal social contacts and networking are important, because sales professionals develop crucial relationships in such encounters. Managers use the knowledge gained in these interactions to help their own organization better meet the needs of customer organizations. In HR selection and hiring processes, as well as in training and daily supervision, research into the function of the amygdala, OT, and other regions of the brain, genes, and hormones in SBRs can aid the study of how well people interact and enjoy social relationships.

Next, we found that SBRs were also better connected for carriers of the *OXTR* GG, leading us to suggest that as salespeople have more contact with people (social wanting), they also train their SBRs to regulate such relationships [a point of view suggested by Chevallier et al. (2012)]. Of course, it also could be the opposite: people who are better at social contact might become better in having/seeking friends, which in turn boosts their liking to be included socially. More research is needed on this topic, but our findings suggest that such questions are worth exploring. Training the SBR involves, in particular, changes in the connectivity of the amygdala with others parts of the SBR (see Bethlehem et al., 2013). In this regard, it is worth mentioning that Bickart et al. (2010) found that the size of people's amygdala correlated with the size of their social network. This might imply that salespeople who are more CO or socially engaged will be better at updating changes in the emotional valences of customers' reactions, which in turn might signal implicit needs of customers. That is, customers cannot always, or might not like to, express their deeper needs, yet well-trained salespeople might learn to read the pain and resistance of customers. Then we could ask the question, could managers train their salespeople to become better in feeling the pain of a customer? Role-play and coaching by experts would be good techniques to do this in the business world. However, the genetic association we found might constrain the effects of training of empathic capabilities of salespeople (for further research on empathy and theory of mind processes in sales professionals, see Bagozzi et al., 2012, 2013). The increased physical size and functional role of the amygdala we found is somewhat analogous to increase in size of the hippocampus found in London Taxi drivers (Maguire et al., 2000).

In summary, our research shows that salespeople with a CO display higher wanting for interactions with customers (meaning they want to build relationships), but equally, they also are keen on observing nuances and resonating with customer moods (social salience). In a related study, we asked 1200 customers whether they identified with CO, as opposed to SO, salespeople. Customers identified more with their CO salespeople and were more willing to stay loyal to them: salespeople with COs were, in other words, perceived enjoyable and convivial by customers, thereby strengthening mutual bonds. Using hyperscanning (Cui et al., 2012), we could study research questions such as: Would customers also mimic salespeople who are carriers of the *OXTR* GG vs. AG/AA alleles? Would customers with *OXTR* GG or AA/AG alleles interact differently/more easily with salespeople who are carriers *OXTR* GG or GA/AA?

It is worth noting that the stimuli in our study were not business stimuli but bare emotional pictures (no contextual information was provided as to specific organizations). This was done for scientific control purposes and helped us more precisely identify clean emotional provocations. A question for future study is—Would carriers of the AA/AG respond differently in terms of connectivity when they view pictures of customers with whom they are asked develop short vs. long-term business relationships, hence manipulating the context (Bartz et al., 2011)? Another question we could ask is, given that CO in our research was self-reported by the salesperson, would similar findings arise if customers themselves make these assessments about the salesperson's interactions (see Kogan et al., 2011 where carriers of the *OXTR* AA vs. AG/GG their social behavior were observed by outsiders)? OT is produced in response to social stimuli and the fluctuations (e.g., for tend/befriend vs. flight/fight or peaceful vs. threatening) in the social environment affect neuropeptide production (Insel, 2010). Thus, questions could be asked such as would carriers of the *OXTR* GG be more patient when confronted with a complaining customer? Alternatively, using blood OT tests, it is possible to gauge OT levels when salespeople actually interact and help customers. Finally, OT administration has been especially beneficial for people low on (self-rated) social proficiency, but not for those high on social proficiency (Bartz et al., 2010). It would be interesting to explore whether OT administered to salespeople who are carriers of the *OXTR* AA/AG allele, as opposed to the GG allele, would benefit more (or equally) in social salience when actually interacting with customers or when wanting to feel socially embedded with customers.

Beyond the research opportunities noted above, some concerns should be mentioned. The sample size in both study 1 and 2 was relatively small, and our findings need to be confirmed (Green et al., 2008). As access to genetic information becomes more possible and affordable, we may be able to study these phenomena using larger data sets in the near future. Using biomarkers (e.g., from genetics, fMRI, or endocrinology) ultimately allows people (managers and employees alike) to better understand the phenotype in question and might also help people develop themselves in more productive and psychologically healthy ways, where biomarkers might function as feedback mechanisms. In addition, of course, if research on OT continues and more evidence becomes available on a (positive) association between *OXTR* and willingness to help customers, genetic information could be used select salespeople. With this growing body of knowledge on how OT affects people's social behavior, this looks promising. However, more research (and consensus) is needed before such biomarkers can actually be used in practice. We also should be aware of the ethical implications of such endeavors.

Our study used only a few biomarkers (connectivity in the brain and genetic markers) that were conceived as indicators for neural and hormonal processes. However, as Insel (2010) importantly suggested, these neural and endocrine processes could well be considered as “dark matter,” meaning that they represent a vast amount of integrative circuits, which remain to be described. In their excellent overviews on OT and its functioning in the brain, Landgraf and Neumann (2004) and Bos et al. (2012) suggested that we should be aware that OT, like other neuropeptides, has complex and dynamic communication patterns involving both synaptic and non-synaptic processes operating at different time frames and with different intensities reaching different brain nuclei and affecting other hormones in diffuse ways. In addition, the OT receptor is itself complex, as is its functioning in different brain nuclei (e.g., Zingg and Laporte, 2003). We did not mention that steroid production affects neuropeptide production, making concrete descriptions on how OT operates at the molecular levels even more complex, which was beyond the scope of our research (e.g., Bos et al., 2012).

### Conflict of interest statement

The authors declare that the research was conducted in the absence of any commercial or financial relationships that could be construed as a potential conflict of interest.

## Appendix

Items from Sales Orientation-Customer Orientation Scale Used in Study 1 [adapted from Saxe and Weitz (1982)].

**Table:**

**Customer orientation**	
1.	I try to get customers to discuss their needs with me.
2.	I try to find out what kind of product would be most helpful to a customer.
3.	I try to bring a customer with a problem together with a product that helps him solve the problem.
4.	I try to give customers an accurate expectation of what the product will do for them.
5.	I try to figure out what a customer's needs are.
**Sales orientation**	
1.	I try to sell a customer all I can convince him to buy, even if I think it is more than a wise customer would buy.
2.	I try to sell as much as I can rather than to satisfy a customer.
3.	If I am not sure a product is right for a customer, I will still apply pressure to get him to buy.
4.	I paint too rosy a picture of my products, to make them sound as good as possible.
5.	It is necessary to stretch the truth in describing a product to a customer.
